# Cutting Edge Research? Realistic Expectations of Priorities, Scope and Engagement: Comment on "‘We’re Not Providing the Best Care If We Are Not on the Cutting Edge of Research’: A Research Impact Evaluation at a Regional Australian Hospital and Health Service"

**DOI:** 10.34172/ijhpm.2023.7792

**Published:** 2023-06-07

**Authors:** Siân Williams, Genevie Fernandes

**Affiliations:** ^1^Healthcare Consultant, London, UK; ^2^Usher Institute, University of Edinburgh, Edinburgh, UK

**Keywords:** Healthcare Research, Impact Evaluation, Stakeholder Engagement, Australia, Research Prioritisation, Health Systems Evidence

## Abstract

While research is linked with informed decision-making and improved healthcare delivery and patient outcomes, the process of generating and translating research evidence in practice and capturing its impact can often be challenging. Based on document and database reviews and interviews in a regional Australian health system, Brown et al discuss the challenges of assessing the impact of research investments over a ten-year period. This commentary explores three inter-related lessons from this article for developing and sustaining a research culture and supporting translation in a health system: (*i*) achieving a shared definition and expectation of research; (*ii*) the importance of stakeholder engagement particularly for research prioritisation; and (*iii*) enabling research across a system. In doing so, it highlights the role and value of engaging knowledge generators and end-users from clinical, management and community domains not only in research development but most importantly in research prioritisation.

 Healthcare research embedded in health services has the potential to improvedecision-making at policy, service and patient level leading to improved quality and outcomes. The article by Brown et al^1^ assesses the impacts of investment in research across an Australian health authority serving a mixed rural and urban population of 250 000 including a tertiary hospital, secondary and primary care services employing over 6000 staff. Using a realist mixed methods approach, it evaluates the outcomes of increased clinician engagement in research and bringing research closer to clinical practice over a ten-year period and explores the challenges of sustaining a research culture and practice. Realist evaluation uses methods to answer the question about “what works for whom in what circumstances” rather than merely “does it work.” Realist evaluators aim to identify the underlying generative mechanisms that explain ‘how’ the outcomes were caused and the influence of context.^2^ Although realist evaluation seems to be an appropriate approach in this study and the authors do present contextual enablers and barriers; the paper could have benefited from details or examples of the actual impacts and the pathways or links between the contextual factors and the impacts.

 Brown and colleagues use an inclusive definition of research that involves a wide variety of inputs: all studies requiring ethical review, implementation focused research, quality improvement and clinical audits. The findings suggest that impact remains challenging to measure and sustainability is not yet secured. They raise attention to the need for system-wide support for research such as dedicated time and backfill, and other incentives as well as a systematic process of data collection to track research investments and evaluate impact. So, what are the wider lessons from this in-depth study? We identify three, which are inter-related: achieving a shared definition and expectation of research; the importance of stakeholder engagement particularly for research prioritisation; and enabling research across a system.

## Shared Definition and Expectation of Research

 Achieving a shared definition and expectation of research calls for complete organisational clarity about its mission and purpose, as well as who is included in the research and where it is needed. Defining these aspects in the health system’s research strategy is the collaborative role of the system leaders, requiring a consultative and participatory approach.

## What Is the Mission?

 The study, including the quotation in its title “We’re Not Providing the Best Care If We Are Not on the Cutting Edge of Research,” suggests that a number of unhelpful assumptions can develop in a health system if there is not a common understanding of the purpose, scope and potential impact of research. Interviews with key informants revealed a range of expectations from research including income generation, improved recruitment and retention of clinical staff, as well as improved patient outcomes. Such diversity in expectation may make it hard to set up sustainable processes to deliver and also to evaluate the impact of the research. A single mission for research is thus required, focused on the desired changes it can bring about. One powerfully argued proposition is for research and the production of knowledge to improve equity.^3^ Certainly, atlases of variation illustrate the distance still to travel to reduce unwarranted variation in healthcare utilisation and outcomes and to improve access for all to evidence-informed care.^4^ A broader mission might be to improve value of healthcare where value has four pillars: personal value (what matters to the individual patient), technical value (doing things right to achieve best possible outcomes with available resources), allocative value (equitable distribution of resources across all patient groups), and societal value (which depends on that society, but could be equity, or societal cohesion).^5^

## What Is the Purpose?

 Once the mission is clear, then it becomes necessary to agree the purpose of any specific research. The World Health Organization (WHO) proposes two distinct but interacting purposes of research: to generate evidence to inform decision-making, and secondly, to apply the evidence.^6^ Further, important questions regard the ambition for the research: at what geographic level is the desired change? The research that is most likely to make a difference to healthcare is that which produces knowledge that can be used on the frontline, especially in primary care settings, in the short term through incremental shifts in behaviour, and expectations should be aligned to this reality. The production of global evidence, typically contributing to systematic reviews or global guidelines; and national or sub-national evidence contributing to a change in national policy, will be of less value to most local health systems, which need actionable evidence to incrementally improve day-to-day decisions about healthcare delivery.^4,6^

## Who Can Do Research?

 Brown et al^1^ found that research was mainly conducted by clinicians, but do other stakeholders have a role? The WHO offers a categorisation of types of research which helpfully opens up the opportunity for a wide range of stakeholders to contribute: scientific codified evidence, tacit colloquial evidence, global and local.^6^ The examples cited in this study illustrate three if not all four of these and it may help manage expectations and engagement if there was explicit acknowledgement of the types of evidence and stakeholders included in the system research strategy. In addition, real health service change should take account of local assets such as the people who Abimbola^3^ calls the “emancipators” (activated citizens and change agents) and the “plumbers” (those who use and produce knowledge day-to-day), as well as local facilities and historical investment.

## When Is the Knowledge Gap a Matter of Education Not Research?

 Of course, not all knowledge needs to be generated from new research. A recent systematic review of translating research evidence into clinical practice showed that the majority of barriers were not mainly organisational but individual skills — in critical appraisal for example.^7^ This implies that an educational strategy about the use of current knowledge must be included as a foundational approach in any health system, and not just reserved for a sub-group who may gain access to university courses or programmes. As Senge^8^ says “sharing knowledge occurs when people [who] are genuinely interested in helping one another develop new capacities for action; it is about creating learning processes.” A Johari window might offer a simple and effective way for system leaders to consider whose knowledge is missing and what strategies might be adopted to address these gaps (see Figure).

**Table:** Adapted Johari Window Using Respiratory Care Examplesto Inform System Research and Education Strategy^9,10^

	Known to me/us*	Not Known to me/us*
Known to others	Common knowledge *(Response: No action required)*	“Blind spot” eg, Oxygen is being used to treat breathlessness without hypoxia (*Response: Education, coaching, learning processes*)
Not known to others	Our unshared knowledge eg, A handheld battery-operated fan can reduce the sense of breathlessness (*Response: Processes for sharing learning such as an audit, journal club*)	Unknown areas for *research* eg, why are women are more likely to get asthma? How do we segment and treat post COVID-19? *(Response: Research study)*

* Us – can be us as researchers, or us as community, or us as policy-makers, or us as service providers.

## Importance of Stakeholder Engagement Particularly for Priority Setting

 Multiple guidance from research funders and agencies such as the WHO highlight the value of engaging stakeholders in research if it is to be sustained.^6,11^ Where engagement is embedded in research, district or national health ministry officials, patients and clinicians are typically identified as the three important stakeholder groups for supporting and influencing the generation and translation of evidence into practice. However, the stakeholders who are often missing are healthcare managers at ward, department or institutional level, who are often ultimately accountable for delivery of healthcare within finite resources. As the authors heard, “* A reason probably why it [clinical practice change] doesn’t happen so much is that people in this hospital are really afraid about accountability […]. You’ve got a great project and you can change practice, if we do this, and if something happens, who’s accountable? And a lot of people can’t answer that question, and when you can’t answer it, nothing happens.” * The study also found it hard to engage managers in the impact assessment. Therefore, it is critically important that the management voice is heard at the start, before research priorities are agreed. The best likelihood of sustained interest and commitment, and evidence application is when there is common agreement amongst all stakeholders about priority candidates for research that align with the larger mission.

 If we look through the lens of health service management and apply the Pareto Principle to consumption of health resources, we should know the most frequent, costly, and variable diagnoses in the hospital and community settings. For example, pre-COVID-19 in the United States, excluding maternal and neonatal inpatient stays, the five most frequent principal diagnoses for hospitalisations in 2018 were septicaemia, heart failure, osteoarthritis, pneumonia (except that caused by tuberculosis), and diabetes mellitus with complication. For each of these the rate of stay per 100 000 population was highest in rural areas. Mental and/or substance use disorder diagnoses ranked among the top five principal diagnoses for people under age 45 years, and for those 45 years and older, the highest rankings were for cardiovascular and musculoskeletal diagnoses. That is, many conditions are flare-ups of chronic disease, demonstrating the importance of research that spans the pathway from the community to the hospital and back again, and includes preventive public health interventions.

 “Cutting edge” research might imply to some stakeholders the development of novel medicines. Yet, we probably have most of the medicines we need to respond to the majority of diseases; most studies conclude that fewer than 15% of drugs approved since the 1970s have real advances over existing drugs.^12^ The new breakthrough drugs such as Ebola and COVID-19 vaccines show that this may be setting dependent and new infections will always warrant drug development responses, but also investment in rehabilitation research which is likely to need pan-national strategies.

 Apart from managers, engaging community stakeholders in research prioritisation is equally valuable: to understand their knowledge and knowledge gaps about these major health challenges and how these can be integrated, for example knowledge about supported self-management. This kind of engagement is aptly illustrated in the reported co-production of research on pain management with the community including Aboriginal and Torres Strait Islander clients. Whilst engagement of multiple stakeholders with unequal knowledge may feel extremely challenging, rapid prioritisation processes appear to be feasible^13^ and delivery of patient-centred outcomes depends on an ongoing dialogue with the community about rights and responsibilities. For example, research is also now considering carbon cost which creates further challenges for health systems to find the right balance between health outcomes, financial and carbon cost.^14^ Such challenges will be best addressed by engaging multiple viewpoints from the start but continuing through all phases of research, including the delivery, analysis, and scaling up of successful interventions. With training and collaboration, multiple stakeholders can also play a role at each phase to achieve engaged scholarship, a demand-driven approach focusing on the research needs of knowledge users.^15^

 Engagement with key stakeholders should not stop inclusion of other research questions if there is a transparent decision-making process in place, because in the short – medium term, funding opportunities may not be aligned with local priorities. Longer term, there is an important role for system leaders to contribute to the debate about research priorities with funders. Lessons from other approaches to collaborations between health systems and academic units also suggest that a strong declaration of interest process must be instituted to avoid risks of bias, particularly where industry financing is available.^16^

## Enabling Research Across a System

 The article recommends investment in infrastructure to improve the sustainability of research and translation of evidence including protected time, access to training, and research management support. Pushing this further, the infrastructure should support a whole-system strategic approach to research prioritisation based on a shared understanding of the mission and purpose. It should enable the early and consistent engagement of stakeholders, particularly priority end users, who may be the “emancipators” (activated citizens) and “plumbers” (users of knowledge) rather than the “engineers” (policy designers) and “professors” (knowledge mongers).^3^ This approach requires a learning system that allows individuals from all clinical and management disciplines across primary, secondary and tertiary care to develop their individual skills in research such as critical appraisal, community engagement, and also analysis of financial cost, health utilisation data, and in, the future, carbon cost. Investment in seamless data collection platforms to routinely track research outputs, outcomes and impact is crucial. System leaders can champion a positive research culture by encouraging cooperation above competition, enabling research by setting up flexible work practices, offering recognition to researchers, and leading relationship building between stakeholders. Impact indicators need to go beyond academic citations and number of grants towards actual changes in clinical skills and practice, patient satisfaction and outcomes. Health systems should value all their assets, which in rural communities such as the one described in the article, where recruitment of healthcare professionals can be hard, means the community itself.

## Acknowledgements

 The authors thank Professor James P. McCormack from the University of British Columbia for his valuable insights on advancements in drug development, which shaped a segment of this commentary.

## Ethical issues

 Not applicable.

## Competing interests

 Authors declare that they have no competing interests.
